# Influence Analysis and Optimization for Aggregate Morphological Characteristics on High- and Low-Temperature Viscoelasticity of Asphalt Mixtures

**DOI:** 10.3390/ma11102034

**Published:** 2018-10-19

**Authors:** Yongchun Cheng, Wensheng Wang, Jinglin Tao, Meng Xu, Xiaoli Xu, Guirong Ma, Shurong Wang

**Affiliations:** 1 College of Transportation, Jilin University, Changchun 130025, China; chengyc@jlu.edu.cn (Y.C.); magrjlu@163.com (G.M.); wangsrjlu@163.com (S.W.); 2 Jiangxi Transportation Institute, Nanchang 330200, China; 3 Jilin Zhongteng Road & Bridge Construction Co., Ltd., Changchun 130022, China; xumeng2018jlu@163.com (M.X.); xuxiaoli2018jlu@163.com (X.X.)

**Keywords:** asphalt mixture, viscoelastic properties, creep, relaxation, aggregates, morphology

## Abstract

Aggregate is an indispensable raw material for asphalt pavement construction. This study evaluates the influences of aggregate morphological characteristics on the high- and low-temperature viscoelasticity of asphalt mixtures. Based on simplex lattice mixture design (SLD), asphalt mix samples were designed and prepared with the same gradation but three different types of aggregates. Subsequently, three morphological characteristics of aggregate (roundness, perimeter index, and erosion-dilation area ratio) are presented to characterize fine and coarse aggregates. Then based on Burgers viscoelastic model, uniaxial compression static creep test was carried out to analyze the high-temperature viscoelastic properties for asphalt mortar and mixture. Meanwhile fitting Prony series models have been utilized to represent relaxation modulus conversed from creep compliance and the low-temperature relaxation characteristics can be also discussed. The experimental results indicated that morphological characteristics of aggregate, especially fine aggregates, are strongly correlated with the viscoelastic parameters of asphalt mixtures. However, the complex morphological characteristics of aggregates have opposite influences on the high- and low-temperature viscoelastic parameters. Therefore, when considering both high- and low-temperature viscoelastic properties, the aggregate proportion was optimized for the appropriate morphological characteristics, which will provide a reference for asphalt mixture design.

## 1. Introduction

Aggregate is an important raw material and it directly affects the performances of asphalt pavement engineering, which is highly related to the efficiency of asphalt pavement maintenance [1]. Aggregate in asphalt mixtures generally accounts for approximately over 90% by weight, which plays a major role for its stability, durability, and mechanical properties.

Researchers have constantly developed research methods to study the aggregates morphological characteristics. Wilson and Klotz [2] presented a method of measuring angularity using Hough transform for quantitative analysis of aggregate angularity. Kuo et al. [3] used a digital image-analysis method to investigate the coarse aggregates morphologies and quantified the morphological characteristics in an effective way. Masad and Button [4] investigated the angularity and texture of fine aggregates by using erosion-dilation method and form factor, in which angularity is analyzed by high-resolution images and texture is described by low-resolution images. Kuo and Freeman [5] defined three image indices called aspect ratio, angularity, and roughness to characterize overall shape, roundness of corners and particle surface texture of fine aggregates. Wang et al. [6] developed a unified Fourier morphological analysis method for quantifying the aggregate morphological characteristics, including shape, angularity, and surface texture. Hu et al. [7] studied the effects of aggregate particles on internal structure in asphalt concrete and results showed that high-temperature damage is mainly caused by coarse aggregate particles. Xie et al. [8] found that digital image processing technique is useful for evaluating morphological characteristics of fine aggregate among three methods. Xiao et al. [9] studied the fine aggregate morphology through using aggregate image measurement system. Ding et al. [10] proposed a modeling algorithm to capture the realistic shape of aggregates and characterize the granular aggregates mechanical properties accurately. Wang et al. [11] proposed an algorithm for modeling two-dimensional virtual aggregates that is based on the Aggregate Imaging Measurement System and the Particle Flow Code in Two Dimensions.

According to existing research on aggregates morphology evaluation, lots of researchers studied the influences of aggregate morphological characteristics on the performances of asphalt mixtures. Chen et al. [12] demonstrated the effects of morphological characteristics of coarse aggregates on the engineering properties of hot-mix asphalt (HMA) mixtures. Masad et al. [13] addressed the relationship between morphological characteristics of fine aggregate and performances of asphalt mixtures and they found that aggregate texture characteristic had the strongest correlation with rutting resistance of asphalt mixtures. Arasan et al. [14] showed that there is a good correlation between some shape indices of aggregate and volumetric performances of asphalt mixtures. Singh et al. [15] utilized aggregate shape parameters to estimate dynamic modulus of asphalt mixes by establishing a model. Pan et al. [16] investigated the effects of coarse aggregate morphology on the permanent deformation of asphalt mixtures. The permanent deformation showed a best correlation with the surface texture and the angularity and surface texture have significant influences on the permanent deformation. Valdes-Vidal et al. [17] investigated the influence of aggregate physical properties on the mechanical properties of asphalt concretes and the results demonstrated that the morphological characteristics of coarse and fine aggregates have influences on strength and anti-cracking of asphalt concretes. Aragao et al. [18] evaluated the influence of morphological properties of aggregates on the mechanical behavior of bituminous mixtures. Thus, the aggregate morphological characteristics have significantly direct influences on the performances of asphalt mixture.

In addition, asphalt mixture is a typical viscoelastic material with the characteristics of both Hookean elasticity and Newtonian viscosity. Its viscoelastic characteristics are the basis for investigation on pavement cracking and permanent deformation of asphalt mixture [19,20]. In general, the physical quantities, such as creep compliance, relaxation modulus, and so on, are used represent the basic viscoelastic characteristics of asphalt mixtures [21]. Despite extensive researches have been conducted to investigate the influence of aggregate morphological characteristics on performances of asphalt mixture, no much study has been conducted for the viscoelastic characteristics of asphalt mixture.

The objective of this study is to relate the aggregate morphological characteristics to the high- and low-temperature viscoelastic properties of asphalt mixture. For that, an experimental proportion design was designed for asphalt mortars and mixtures were prepared with the same gradation but three different types of aggregates based on simplex lattice design (SLD) method. Three aggregate morphological characteristics (roundness, perimeter index and erosion-dilation area ratio) are chosen to characterize fine and coarse aggregates. Then based on Burgers viscoelastic model, uniaxial compression static creep test was carried out to analyze the high-temperature viscoelastic properties for asphalt mix. Meanwhile, by fitting Prony series models, relaxation modulus can be conversed from creep compliance and the low-temperature relaxation characteristics can be also discussed.

## 2. Materials and Experimental Methods

### 2.1. Raw Materials

In this study, the base asphalt AH-90 obtained from Panjin of Liaoning Province in China was used and its physical properties are listed in Table 1. The mineral filler used is limestone powder, for which the diameter with percent passing 50% is 2.326 µm, and its properties are shown in Table 2. In order to demonstrate the effects of morphological characteristics of aggregates, three different types of coarse and fine aggregates from Jilin Province in China were selected, in which the coarse aggregates include basalt stone, andesite stone, and pebble, and the fine aggregates are basalt manufactured sand, andesite manufactured sand, and river sand. Their physical properties are shown in Table 3 and Table 4.

### 2.2. Sample Preparation and Aggregates Morphological Characteristics

#### 2.2.1. Sample Preparation

(1) Aggregate Proportion Design

Based on the mathematical theory, statistical analysis, and experimental design [22], SLD as a common mixture design method, has been utilized to investigate the influences of the proportions of three mixture components on the viscoelastic characteristics of asphalt mixes using Design-Expert 8.0 software (Stat-Ease Inc., Minneapolis, MN, USA). Three types of aggregates, namely basalt, andesite, and pebble/river sand, are considered as three different components, and the samples of asphalt mixes can be prepared through mixing the three ingredients at different proportions. Then, a larger range of morphological parameters for these aggregates could be designed by using SLD. The designed proportions of three components in asphalt mixes are listed in Table 5, for example, in sample group 1, basalt:andesite:pebble/river sand = 0.167:0.667:0.167.

(2) Asphalt Mortar and Mixture Preparation

**Figure 1:**
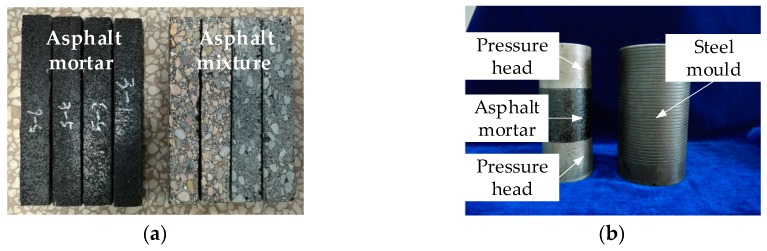
Samples of asphalt mixture and mortar: (**a**) Beam samples; and, (**b**) Cylindrical samples.

In this study, asphalt mixes including mortar and mixture samples were prepared to analyze and demonstrate the influences of morphological characteristics of coarse and fine aggregates on the high- and low-temperature viscoelastic characteristics, respectively. The gradation of asphalt mixture was selected the typical dense gradation with a nominal maximum size of 13.2 mm, i.e., AC-13. Asphalt mortar consists of asphalt, mineral filler and fine aggregate passing through 2.36 mm sieve size, and its gradation was similar to AC-13. The detailed sample preparation process was given in a previous study [23]. After determining asphalt contents, each component content in asphalt mortar and mixture could be calculated according to the densities of aggregate and asphalt, which are listed in Table 6. Then, the cylindrical samples of asphalt mortar with 50 mm in height and 50 mm in diameter were prepared through the static pressure method [24], and the Marshall specimens of asphalt mixture with 63.5 mm in height and 101 mm in diameter were made by Marshall compactor (Lambert Testing Machine Co., Ltd., Tianjin, China) according to the Chinese specification JTG E20-2011 [25]. However, beam samples are generally used for low-temperature property test. Therefore, beam samples with a length of 250 mm, width of 30 mm, and height of 35 mm, were prepared for asphalt mortar by a self-designed mould under static pressure; the square slab with length and width of 300 mm and thickness of 50 mm were prepared and then made into beam samples with 250 mm × 30 mm × 35 mm for asphalt mixture. These samples are shown in Figure 1.

#### 2.2.2. Morphological Characteristics of Aggregates: Shape, Angularity and Texture

The image processing technique is employed to process (image denoising and enhancement) these color images, in which the images of coarse and fine aggregate were obtained by scanner and stereo microscope (Opt Vision Technology Co., Ltd., Dongguan, China). Then, the morphological characteristics of aggregates, including shape, angularity, and texture characteristics, can be obtained according to previous study [23]. Roundness, perimeter index, and erosion-dilation area ratio were used to represent the shape, angularity, and texture characteristics of aggregates, which were defined and could be measured in previous study [23]. The mean values of logarithmic normal distribution were selected to represent aggregate morphological characteristics. *Composite index* for fine and coarse aggregates is adopted to unify and account for these three morphological characteristics, i.e., roundness, perimeter index, and erosion-dilation area ratio. Composite morphological characteristics for fine and coarse aggregates are abbreviated as *FR* and *CR* for roundness, *FPI* and *CPI* for angularity, and *FEDR* and *CEDR* for texture, respectively. The results of *Composite index* for fine aggregate in asphalt mortar (abbreviated as F1~F10) and coarse aggregate in asphalt mixture (abbreviated as C1~C10) are listed in Table 7.

### 2.3. Experimental Methods

#### 2.3.1. High-Temperature Viscoelastic Property Tests

**Figure 2:**
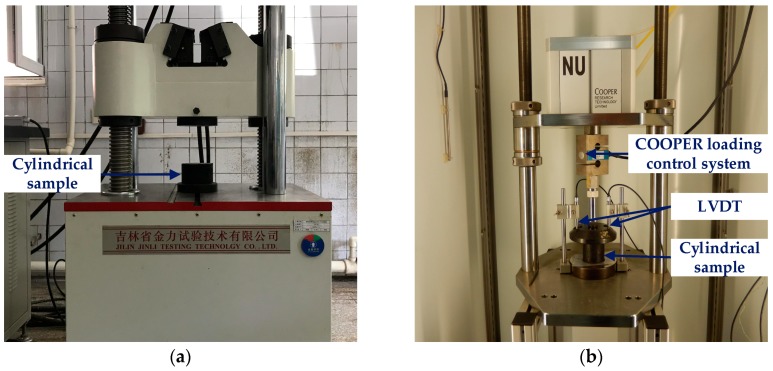
High-temperature viscoelastic property tests: (**a**) Uniaxial compression failure test; and, (**b**) Uniaxial compression static creep test.

Uniaxial compression failure test (Jinli testing technology Co., Ltd., Changchun, China) and uniaxial compression static creep test (Cooper Research Technology Ltd., Ripley, UK) were adopted to study the high-temperature viscoelastic characteristics of asphalt mixes and they were conducted in accordance with previous study [23,26,27,28]. Due to different asphalt contents in asphalt mortar and mixture, three test temperatures were selected to reduce the error caused by temperature, in which the test temperatures were set as 10 °C, 20 °C, and 30 °C for asphalt mortar and 30 °C, 40 °C and 50 °C for asphalt mixture. Uniaxial compression failure test is shown in Figure 2a and a servo-pneumatic universal testing machine, as shown in Figure 2b, was employed to conduct the uniaxial compression static creep test at a fixed stress level and creep time for asphalt mixes.

The stress level is *σ* = *σ*_0_, the creep compliance of asphalt mortar and mixture is defined as follows:

*J*(*t*) = *ε*(*t*)/*σ*_0_, (1)

where *J*(*t*) is creep compliance; *ε*(*t*) is creep strain; and, *σ*_0_ is constant stress.

#### 2.3.2. Low-Temperature Viscoelastic Property Tests

(1) Beam Bending Failure Test

**Figure 3:**
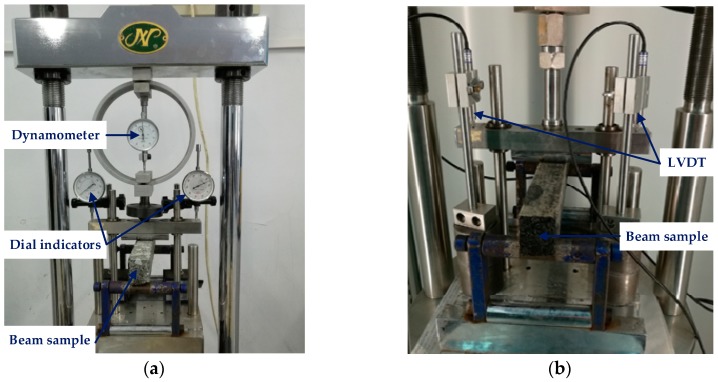
Low-temperature viscoelastic property tests: (**a**) Beam bending failure test; and, (**b**) Beam bending creep test.

Beam bending failure test (Jinli testing technology Co., Ltd., Changchun, China) was used to determine the stress level of the beam bending creep test. The beam samples of asphalt mortar and mixture were placed in an environmental chamber for at least 4 h and the test temperature is −5 °C. Then a concentrated load was applied at the middle span of the beam sample until the beam was broken, which is shown in Figure 3a. The applied load and corresponding vertical deformation at the middle span were recorded, therefore, the failure stress, failure strain, and stiffness modulus could be calculated by using the following equations.

*R_B_* = (3 × *L* × *P_B_*)/(2 × *b* × *h*^2^), (2)

*ε_B_* = (6 × *h* × *d*)/*L*^2^, (3)

*S_B_* = *R_B_/ε_B_*, (4)

where *R_B_*, *ε_B_*, *S_B_* are the failure stress, failure strain and stiffness modulus, respectively; *b*, *h*, *L* are the width, height, and length of the test beam sample, respectively; *P_B_* is the failure load; and, *d* is the failure deformation at the middle span.

(2) Beam Bending Creep Test

In the beam bending creep test (Cooper Research Technology Ltd., Ripley, UK), a constant load was applied at the middle span of beam sample and the deformation versus time was recorded to evaluate the creep property of asphalt mortar and mixture. The test procedure is similar to uniaxial compression static creep test and the beam bending creep test is shown in Figure 3b. Then, the strain versus time in the beam bending creep could be obtained and the creep compliance of asphalt mortar and mixture was also calculated by Equation (1).

## 3. Results and Analysis for High-Temperature Creep Properties of Asphalt Mixes

A Burgers model is developed as a combination of Maxwell and Kelvin models in series, which is a four-element model indicating elastic deformation, viscous flow, and viscoelastic deformation [29,30]. And the Burgers model used in this study has been described in details [23]. For the Burgers model, *E*_1_, *E*_2_, *η*_1_, *η*_2_ are viscoelastic constants, which could be determined through the fitting creep test. *E*_1_ is the modulus of immediate elasticity Burgers model, *E*_2_ is the modulus of delayed elasticity Burgers model, *η*_1_ is the coefficient of viscosity Burgers model, *η*_2_ is the coefficient of elastic delay viscosity Burgers model and *τ* = *η*_2_/*E*_2_ is defined as the retardation time.

### 3.1. Analysis for Fine Aggregate Morphological Characteristics on Creep Properties of Asphalt Mortar

#### 3.1.1. Uniaxial Compression Failure Test Results of Asphalt Mortar

**Figure 4:**
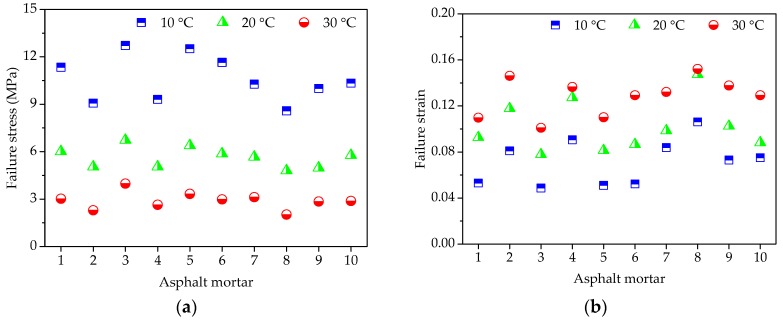

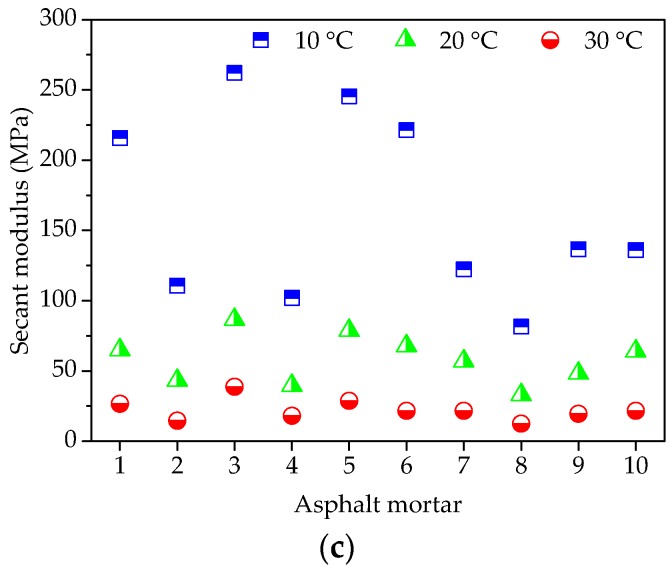
Failure results of asphalt mortars: (**a**) Failure stress; (**b**) Failure strain; and, (**c**) Secant modulus.

For uniaxial compression failure tests of asphalt mortar, the test temperatures were selected as 10 °C, 20 °C, and 30 °C, respectively, and the applied loading speed was set as 50 mm/min. According to the recorded relation curve between force and displacement, the maximum stress, corresponding strain, and the ratio of two were obtained as the compression failure stress, failure strain and secant modulus. The uniaxial compression failure test results are plotted in Figure 4.

As shown in Figure 4a, asphalt mortars with different morphological characteristics of fine aggregate have obvious different mechanical properties when subjected to the constant strain rate loading. At the same temperature, F3 has the highest failure stress, and followed by F5, the failure stress of F8 is the lowest. A higher failure stress is preferable due to the better bearing capacity. Simultaneously, it could be observed that temperature has a significant influence on failure stress of asphalt mortar. The failure stress of asphalt mortar decreases dramatically with temperature.

As for the failure strain in Figure 4b, it illustrates the deformation characteristics of asphalt mortars while loading, which has an opposite trend with the failure stress in Figure 4a. For any test temperature, F8 has the largest failure strain, whereas the failure strain of F3 is the smallest among all of the asphalt mortars. The failure strain indicates the deformation before asphalt mortar being broken, which could help to reduce the occur of crack at low temperature but would be easy to produce greater permanent deformation at high temperature. In addition, it is clear that the higher the test temperature is, the larger the failure strain of asphalt mortar becomes, which is attributed to the high fluidity of asphalt at higher temperature.

Secant modulus is the ratio of failure stress to failure strain, comprehensively reflecting the compatibility of deformation. Normally, a higher secant modulus stands for the better compatibility of deformation and anti-compression failure performance. The variation trend of secant modulus results that was observed in Figure 4c is consistent with the trend of failure stress in Figure 4a, in which F3 has the highest secant modulus, and followed by F5, the secant modulus of F8 is the lowest among all the asphalt mortars for any test temperature. As the designed experimental proportion listed in Table 5, the asphalt mortar F3 was prepared by andesite manufactured sand, basalt manufactured sand was used for F5 and F8 was made by river sand. Thus, it is evident that manufactured sands can improve the anti-compression failure performance of asphalt mortar and the asphalt mortar made by natural sands has the largest failure strain. Due to asphalt as a typical viscoelastic material, temperature has an obvious effect on its mechanical properties. Asphalt binder has a higher cohesive force and the inlay effect of aggregate is more significant, resulting in a bigger difference among different asphalt mortars at a lower temperature. However, the high fluidity of asphalt at higher temperature causes the cohesive force of asphalt binder to decrease and the aggregate has a weak influence.

#### 3.1.2. Uniaxial Compression Creep Test Results of Asphalt Mortar

(1) Strain Results of High-Temperature Creep Test

**Figure 5:**
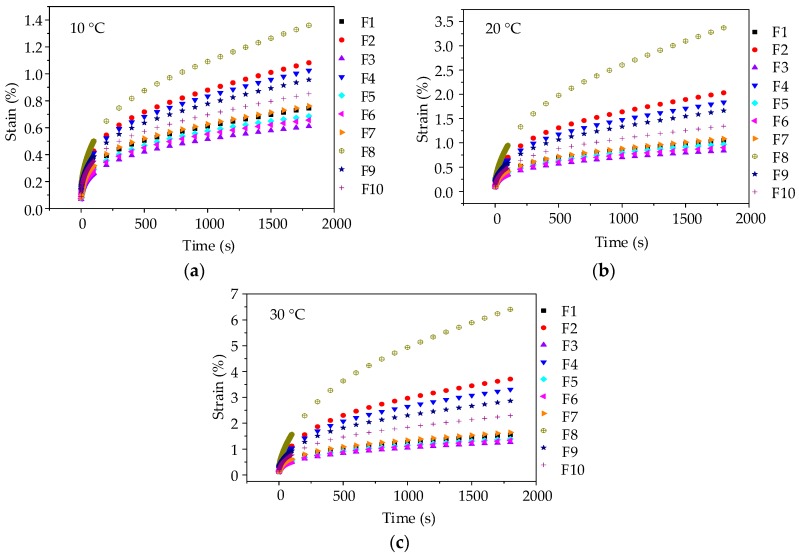
Creep strain-time curves of asphalt mortars: (**a**) 10 °C; (**b**) 20 °C; and, (**c**) 30 °C.

Before each uniaxial compression static creep test, uniaxial compression failure test was conducted to determine an appropriate stress level for asphalt mortar. Figure 4a shows the range of failure stress, i.e., 8.62 MPa~12.74 MPa at 10 °C, 4.83 MPa~6.76 MPa at 20 °C, 2.07 MPa~4.03 MPa at 30 °C. Therefore, the applied stress level of 0.2 MPa was kept constant for the uniaxial compression creep test. Then, a preconditioning stress of 5% loading was applied to asphalt mortar samples for 90 s. Subsequently, a servo-pneumatic universal testing machine was adopted to apply a stress-controlled uniaxial compressive loading for 1800 s at 10 °C, 20 °C, and 30 °C, respectively. Figure 5 compares the creep strain for 10 groups of asphalt mortars at different temperatures.

As illustrated in Figure 5, the creep deformations of asphalt mortars increase gradually with the test temperature and loading time increasing. When the test temperature changes from 10 °C to 30 °C, the creep strain at 1800 s increases 2~4 times, which could be explained by the viscoelastic property of asphalt mortar. Besides, it could be observed that at the same loading time, the creep strains of 10 groups of asphalt mortars at any test temperature are ranked as F8 > F2 > F4 > F9 > F10 > F7 > F1 > F5 > F6 > F3. A larger creep strain means a worse anti-deformation performance, that is, F3 has the best anti-deformation performance and the anti-deformation performance of F8 is the worst. Thus, andesite manufactured sand could improve the anti-deformation performance of asphalt mortar with respect to river sand.

(2) Influence of Fine Aggregate on High-Temperature Viscoelastic Parameters

**Figure 6:**
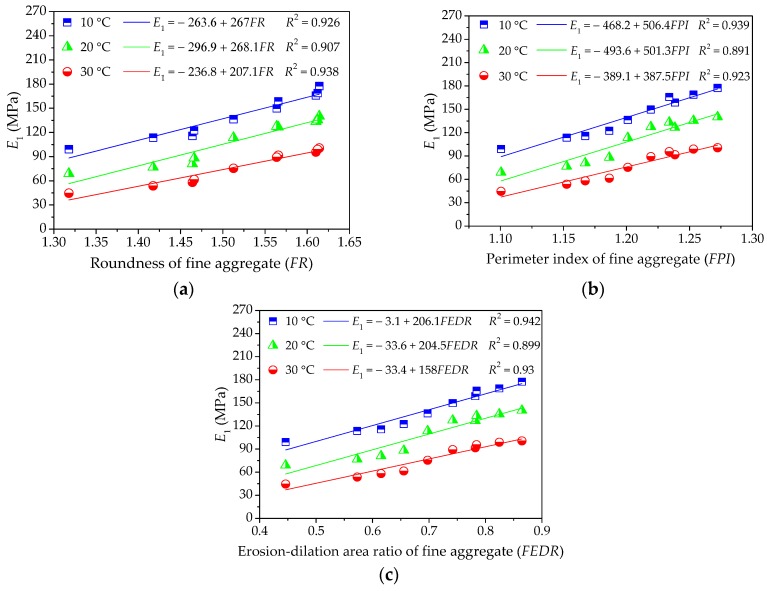
Influences of fine aggregate morphological characteristics of on *E*_1_ of asphalt mortar: (**a**) Shape (*FR*); (**b**) Angularity (*FPI*); and, (**c**) Texture (*FEDR*).

**Figure 7:**
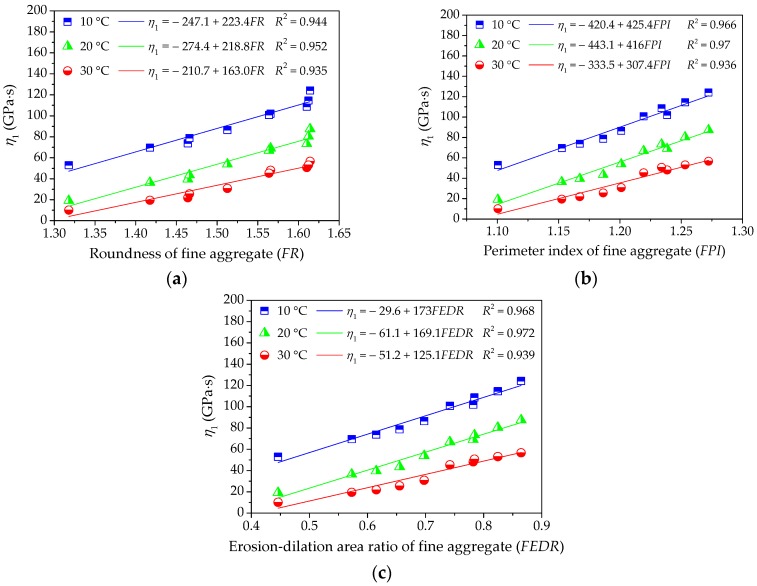
Influences of fine aggregate morphological characteristics of on *η*_1_ of asphalt mortar: (**a**) Shape (*FR*); (**b**) Angularity (*FPI*); and, (**c**) Texture (*FEDR*).

**Figure 8:**
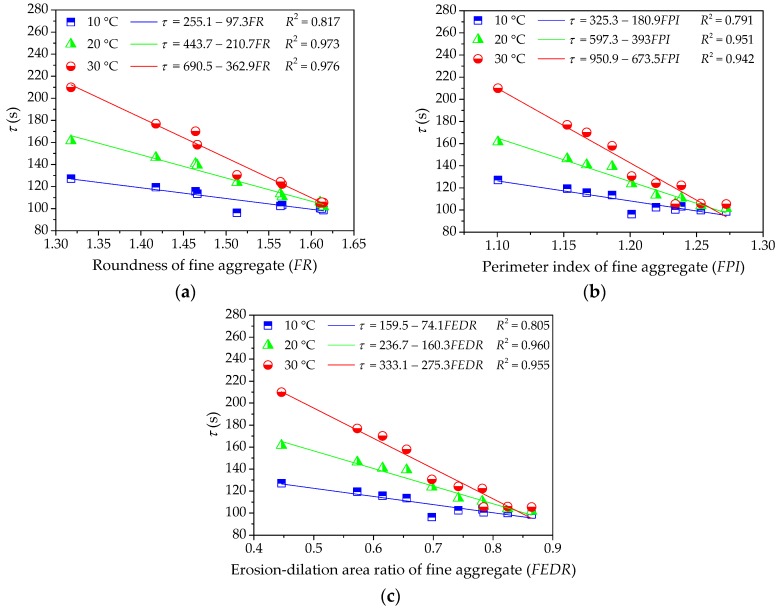
Influences of fine aggregate morphological characteristics of on *τ* of asphalt mortar: (**a**) Shape (*FR*); (**b**) Angularity (*FPI*); and, (**c**) Texture (*FEDR*).

As different fine aggregates lead to different viscoelastic performances of asphalt mortars, it is necessary to quantitatively analyze the influence of morphological characteristics of fine aggregates on viscoelastic performances of asphalt mortar. The relationship between morphological characteristics of fine aggregate and viscoelastic performances of asphalt mortar could be obtained by using linear regression, in which morphological characteristics (i.e., *FR*, *FPI*, and *FEDR*) are regarded as independent variables and viscoelastic parameters (i.e., *E*_1_, *η*_1_, and *τ*) are response variables. These relationship and correlation analysis are presented in Figure 6, Figure 7 and Figure 8.

As shown in Figure 6 and Figure 7, the values of *E*_1_ and *η*_1_ increase with morphological characteristics of fine aggregate increasing. There could be a positive correlation among these parameters and the correlation coefficient values *R*^2^ are above 0.89. The linear regression models show a strong correlation with test results, indicating that the linear regression models are efficient in characterizing their relationship. A higher value of *E*_1_ stands for a larger resistance to deformation while loading as well as a better recovery capacity after unloading, and the larger the viscosity coefficient *η*_1_, the smaller the permanent deformation. Thus, fine aggregate with complex morphological characteristics could improve the anti-deformation performance of asphalt mortar. In addition, the slopes of linear regression models decrease slightly with temperature increasing, which may be more related to the rheological property of asphalt binder.

Figure 8 shows the influence of morphological characteristics of fine aggregate on the retardation time *τ* of asphalt mortar. It clearly shows that the retardation time of asphalt mortar decreases with increase in morphological characteristics of fine aggregates and the slopes of the descending segments increase with temperature. Morphological characteristics of fine aggregate are shown to be strongly correlated with the retardation time of asphalt mortar at lower temperature (all the *R*^2^ values are above 0.9), whereas a relatively small correlation is observed at higher temperature. Due to the retardation time that is related to the recovery time of viscoelastic deformation, fine aggregate with complex morphological characteristics helps to improve the deformation recovery capacity of asphalt mortar.

### 3.2. Analysis for Coarse Aggregate Morphological Characteristics on Creep Properties of Asphalt Mixture

#### 3.2.1. Uniaxial Compression Failure Test Results of Asphalt Mixture

**Figure 9:**
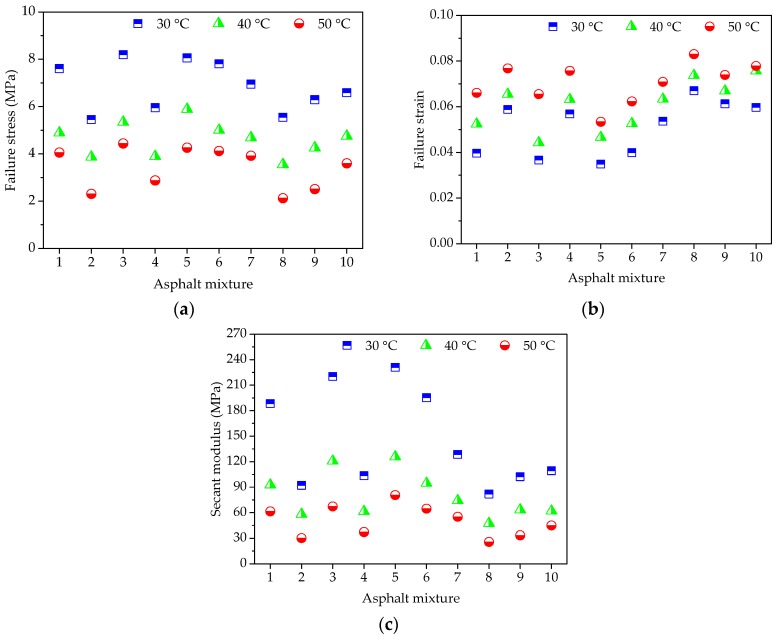
Failure results of asphalt mixtures: (**a**) Failure stress; (**b**) Failure strain; and, (**c**) Secant modulus.

Figure 9 presents the uniaxial compression failure test results of asphalt mixtures at the three test temperatures of 10 °C, 20 °C, and 30 °C. The speed of applied loading was also 50 mm/min. As shown in Figure 9, for constant strain rate loading, different types of coarse aggregate lead to a large difference in mechanical property distribution of asphalt mixtures. At the same temperature, asphalt mixtures without pebbles have larger failure stress and secant modulus and those with pebbles have smaller failure strain, i.e., the failure stress and secant modulus of C3, C5, and C6 are larger than the others, while these failure strains are smaller than the other groups. What’s more, the change of mechanical properties for asphalt mixtures should be more related to the pebble content. This is because manufactured stones (basalt stone and andesite stone) have the more complex morphological characteristics than pebble and pebble is more spherical. With loading time and increasing deformation, the inlay effect of coarse aggregate becomes more and more significant. However, asphalt mixture with pebble would occur cracks prematurely when compared to asphalt mixture made with manufactured stones, resulting in stress reduction as well as broken specimens.

Regarding the influence of temperature on the mechanical properties of asphalt mixtures, it is also consistent with the finding of asphalt mortars. The failure stress and secant modulus of asphalt mixture decrease significantly and the failure strain increases with temperature increasing. These simple observations indicate that asphalt mixture has significant temperature susceptibility, which is due to the viscoelastic property of asphalt.

#### 3.2.2. Uniaxial Compression Creep Test Results of Asphalt Mixture

(1) Strain Results of High-Temperature Creep Test

**Figure 10:**
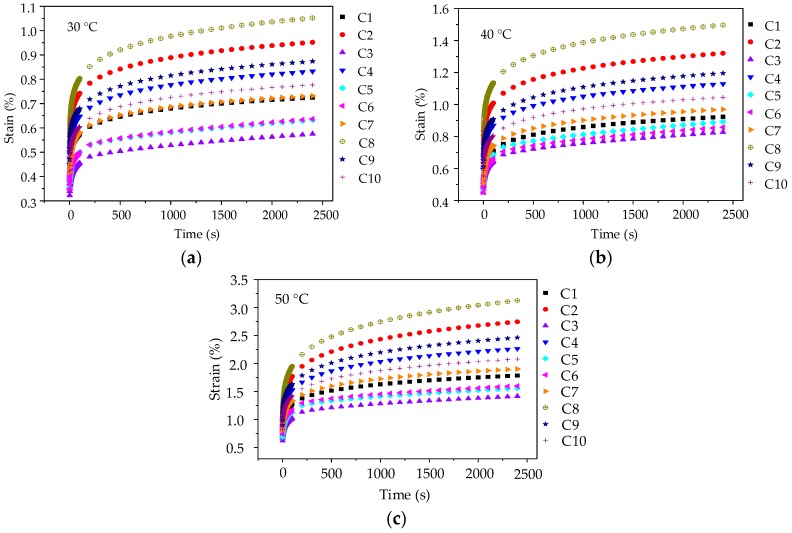
Creep strain-time curves of asphalt mixtures: (**a**) 30 °C; (**b**) 40 °C; and, (**c**) 50 °C.

The range of failure stress for asphalt mixtures are 5.48 MPa~8.21 MPa at 30 °C, 3.55 MPa~5.91 MPa at 40 °C, and 2.13 MPa~4.46 MPa at 50 °C. Thus, the applied stress level of 0.4 MPa was kept constant for the creep test. A preconditioning stress of 5% loading was also applied to asphalt mixture specimens for 90 s. Subsequently, a servo-pneumatic universal testing machine was adopted to apply a stress-controlled uniaxial compressive loading for 2400 s at three test temperatures. The creep strain results for 10 groups of asphalt mixtures are depicted in Figure 10.

As illustrated in Figure 10, it is also observed that the creep deformations of asphalt mixtures increase gradually with test temperature increasing due to the viscoelastic property of asphalt. However, it is worth noting that when compared with the creep curves of asphalt mortars, the creep strains of asphalt mixtures are still lower under the condition that stress level is twice, test temperature is 20 °C higher and creep time is 600 s longer. This observation fully demonstrated that the skeleton effect of coarse aggregate could enhance the anti-deformation performance of asphalt mixture effectively. Besides, it could be also observed that, at the same loading time and test temperature, the anti-deformation performance of asphalt mixture that was prepared by manufactured stones (basalt stone and andesite stone) is better than that of asphalt mixture with pebble, and the creep strains of 10 groups of asphalt mixtures at any test temperature are ranked as C8 > C2 > C9 > C4 > C10 > C7 > C1 > C6 > C5 > C3. Thus, coarse aggregate with complex morphological characteristics could improve the anti-deformation performance of asphalt mixture.

(2) Influence of Coarse Aggregate on High-Temperature Viscoelastic Parameters

**Figure 11:**
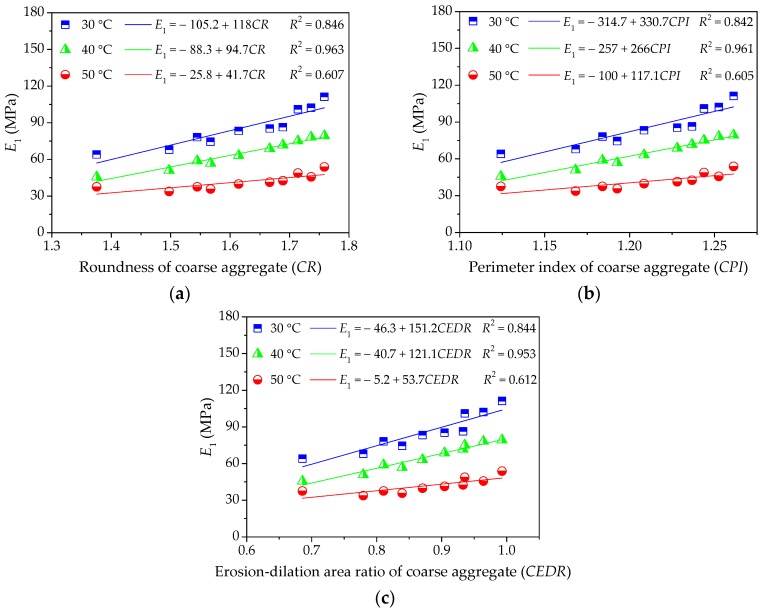
Influences of coarse aggregate morphological characteristics of on *E*_1_ of asphalt mixture: (**a**) Shape (*CR*); (**b**) Angularity (*CPI*); and, (**c**) Texture (*CEDR*).

**Figure 12:**
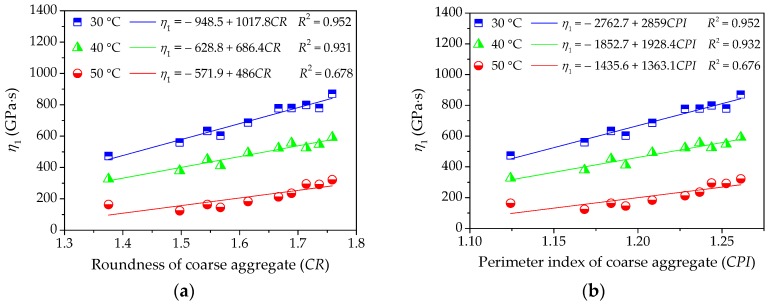

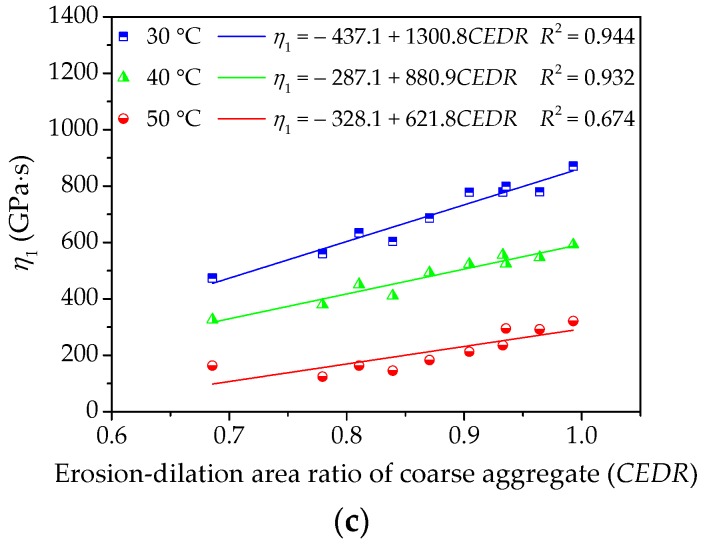
Influences of coarse aggregate morphological characteristics of on *η*_1_ of asphalt mixture: (**a**) Shape (*CR*); (**b**) Angularity (*CPI*); and, (**c**) Texture (*CEDR*).

**Figure 13:**
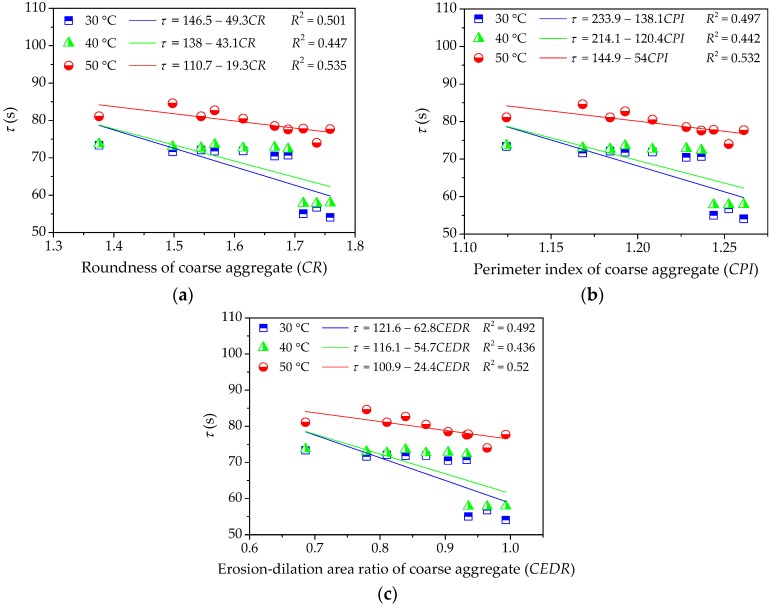
Influences of coarse aggregate morphological characteristics of on *τ* of asphalt mixture: (**a**) Shape (*CR*); (**b**) Angularity (*CPI*); and, (**c**) Texture (*CEDR*).

To further investigate the influence of morphological characteristics of coarse aggregate on the viscoelastic performances of asphalt mixture at a high temperature, the viscoelastic constants in Burgers model can be given by fitting the uniaxial compression creep curve results. Then, this study respectively takes morphological characteristics and viscoelastic parameters as independent variables and response variables and their relationships are depicted in Figure 11, Figure 12 and Figure 13, which is convenient for the quantitative regression analysis.

Figure 11 and Figure 12 clearly show that the modulus *E*_1_ of immediate elasticity Burgers model and coefficient *η*_1_ of viscosity Burgers model increase gradually with morphological characteristics of coarse aggregate. The higher correlation efficient values *R*^2^ indicate that the linear regression models show a strong correlation with test results at a lower temperature. However, the slopes of linear regression models decrease slightly with temperature increasing and the correlation efficient values *R*^2^ also become relatively smaller, which means a relatively insignificant influence of coarse aggregate on *E*_1_ and *η*_1_ and a higher dispersion at higher temperature. Morphological characteristics of coarse aggregate are typically more related to the resistance to deformation while loading as well as the permanent deformation at lower temperature, but they present a relatively less and discretized influence on viscoelastic characteristics of asphalt mixture.

As for the effects of morphological characteristics of coarse aggregate on the retardation time of asphalt mixture, the correlation efficient values *R*^2^ are much lower than 0.54, showing that there is no clear linear correlation between the retardation time of asphalt mixture and morphological characteristics of coarse aggregates. In fact, the retardation time of asphalt mixtures generally decreases with increase in morphological characteristics. This is expected because the viscoelastic deformation in the creep process mainly occurs in asphalt mortar, which plays the roles of bonding with coarse aggregates and filling internal voids. Hence, coarse aggregates are not as strongly correlated with the retardation time as fine aggregates.

## 4. Results and Analysis for Low-Temperature Relaxation Properties of Asphalt Mixes

### 4.1. Conversion between Relaxation Modulus and Creep Compliance

In low-temperature relaxation analysis, it is necessary to convert the creep compliance into the relaxation modulus [21,31]. The creep compliance in time domain for the asphalt mixture can be formulated in the form of Prony series:

Taking the inverse Laplace transform, the creep strain versus time is given by:

(5) $$J(t)=J_{0}+\frac{t}{\eta }+\sum_{i=1}^{n}J_{i}\left(1-e^{-t/\tau_{i}}\right),$$

where *J*(*t*), *J*_0_ and *η* are Prony series parameters, *τ_i_* is the retardation time.

Applying the Laplace transform gives

(6) $$\hat{J}(s)=\frac{J_{0}}{s}+\frac{1}{\eta \cdot s^{2}}+\sum_{i=1}^{n}\frac{J_{i}}{s(\tau_{i}\cdot s+1)},$$

A relationship between $\hat{E}(s)$ and $\hat{J}(s)$ is presented as:

(7) $$\hat{E}(s)\hat{J}(s)=\frac{1}{s^{2}},$$

Thus, it yields

(8) $$\hat{E}(s)=\frac{1}{s(J_{0}+\frac{1}{\eta \cdot s}+\sum_{i=1}^{n}\frac{J_{i}}{\tau_{i}\cdot s+1})},$$

Equation (8) could be rewritten as the polynomial form:

(9) $$\hat{E}(s)=\frac{\left(a_{n}\cdot s^{n}+a_{n-1}\cdot s^{n-1}+\cdots +a_{1}\right)}{\left(b_{n+1}\cdot s^{n+1}+b_{n}\cdot s^{n}+\cdots +b_{1}\right)},$$

where *a* and *b* are the coefficients of polynomial function. Then, Equation (9) is expanded using the factorization as:

(10) $$\bar{E}(s)=\frac{C_{1}}{s+\frac{1}{\lambda_{1}}}+\frac{C_{2}}{s+\frac{1}{\lambda_{2}}}+\cdots +\frac{C_{n}}{s+\frac{1}{\lambda_{n}}},$$

where 1/*λ*_1_, 1/*λ*_2_ and 1/*λ_n_* are the roots of denominator polynomial; *C*_1_, *C*_2_, and *C_n_* are the values for decomposing polynomial.

Applying the inverse Laplace transform to Equation (10) leads to the relaxation modulus of the generalized Maxwell model with an individual spring element:

(11) $$E(t)=E_{\infty }+\sum_{i=1}^{n}E_{i}e^{-t/\rho_{i}},$$

where *E*_∞_ is the equilibrium modulus; *E_i_* is the elastic modulus of the *i*th Maxwell element and *ρ_i_* is the relaxation time of the *i*th Maxwell element; and, *n* is the number of Maxwell element.

### 4.2. Analysis for Fine Aggregate Morphological Characteristics on Relaxation Properties of Asphalt Mortar

#### 4.2.1. Beam Bending Failure Test Results of Asphalt Mortar

**Figure 14:**
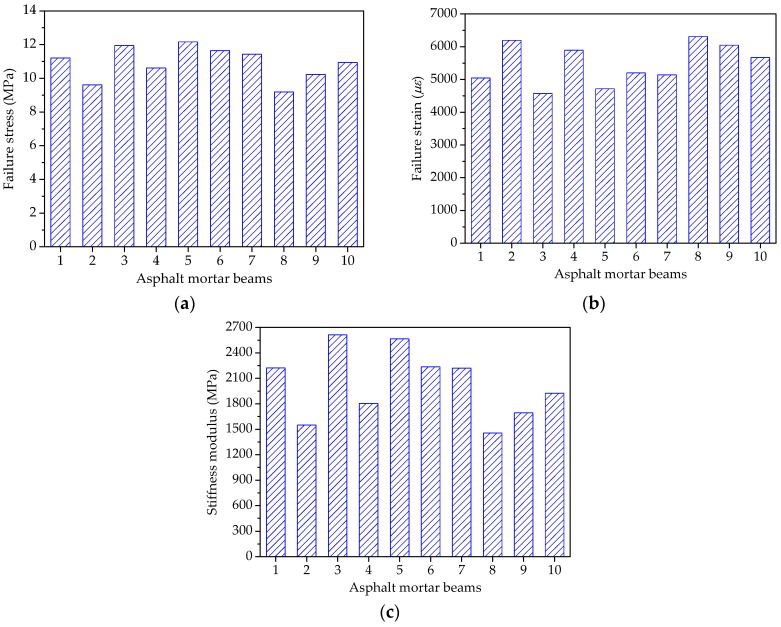
Failure results of asphalt mortar beams: (**a**) Failure stress; (**b**) Failure strain; and, (**c**) Stiffness modulus.

The test temperature was −5 °C and the speed of applied loading was 50 mm/min in the beam bending failure test of asphalt mortar to determine an appropriate stress level for further beam bending creep test. The beam specimens of asphalt mortars were placed in an environmental chamber for at least 4 h and the beam bending failure test results could be expressed as the average of three replicates for each group of asphalt mortars. Figure 14 shows the bending failure stress, bending failure strain, and stiffness modulus results of all the asphalt mortars.

As shown in Figure 14a, asphalt mortars with different morphological characteristics of fine aggregate have obvious different ultimate loads in the beam bending failure test. The bending failure stress could be ranked from big to small, as follows: F5 > F3 > F6 > F7 > F1 > F10 > F4 > F9 > F2 > F8. The bigger the bending failure stress, the more the ultimate load and the better the bending failure performance. Therefore, due to the better cohesive effect between fine aggregate with complex morphological characteristics and asphalt binder, it is evident that manufactured sands can improve the bending failure strength of asphalt mortar.

Figure 14b compares the maximum deformation of asphalt mortars in the beam bending failure test. The bending failure strains for 10 groups of asphalt mortars are ranked as F8 > F2 > F9 > F4 > F10 > F6 > F7 > F1 > F5 > F3. A higher bending failure strain is preferable for the low-temperature cracking resistance, which indicates that the asphalt mortar made by manufactured sands is easier to produce the low-temperature embrittlement. This is because fine aggregate with complex morphological characteristics would occur the stress concentration phenomenon while loading, leading to cracks at stress concentration points.

As for the stiffness modulus in Figure 14c, stiffness modulus characterizes the compatibility of deformation, comprehensively reflecting the relationship between load and deformation. Figure 14c clearly shows that the stiffness modulus can be ranked as: F3 > F5 > F6 > F1 > F7 > F10 > F4 > F9 > F2 > F8. Asphalt mortar prepared by river sand has the smallest stiffness modulus, suggesting that river sand could improve the low-temperature cracking resistance of the asphalt mortar.

#### 4.2.2. Beam Bending Creep Test Results of Asphalt Mortar

(1) Strain Results of Low-Temperature Creep Test

**Figure 15:**
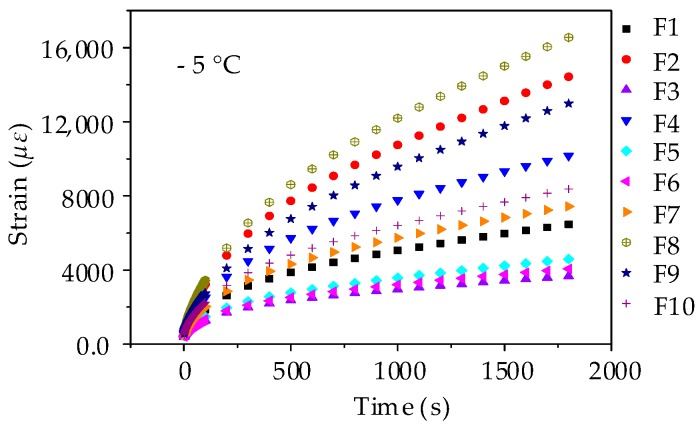
Creep strain-time curves of asphalt mortar beams.

A servo-pneumatic universal testing machine was used to conduct the low-temperature creep test at −5 °C for 1800 s. The bending stress level was set as 1.38 MPa for the stress-controlled uniaxial compressive loading. Based on the measured test results, Figure 15 depicts the bending strains against the loading time in the beam bending creep test. It clearly shows that the bending strains of beam samples increase gradually with the loading time and the creep strains for 10 testing groups are ranked as F8 > F2 > F9 > F4 > F10 > F7 > F1 > F5 > F6 > F3. Asphalt mortar that is prepared by river sand (i.e., F8) has the largest strain, whereas asphalt mortar prepared by andesite manufactured sand (i.e., F3) has the smallest strain. What is more, the bending creep strain exhibits a rising trend with the content of river sand. This observation is consistent with the morphological analysis that, due to river sands close to spherical, the more river sands asphalt mortar has, the weaker the inlay effect of aggregates.

**Figure 16:**
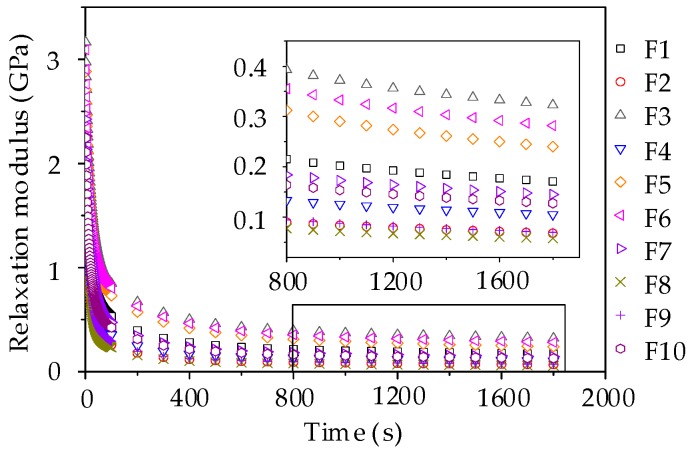
Relaxation modulus curve of asphalt mortars.

The bending creep strain curves in Figure 15 were used to determine the creep compliance by using the fitting method. Then, based on the conversion between the relaxation modulus and creep compliance, the Prony series model of relaxation modulus could be conducted for 10 groups of asphalt mortars. In general, the relaxation time is on an axis using the logarithmic scale and the relaxation time are selected as 0.5 s, 5 s, 50 s, 500 s, and 5000 s. Table 8 summarizes the parameters of Prony series model of relaxation modulus and the *R*^2^ values for asphalt mortars. It is found that the Prony series model can provide excellent fittings with *R*^2^ values larger than 0.998, indicating that this Prony series model is able to accurately describe the relaxation characteristics of asphalt mortars. Subsequently, the constructed Prony series models were utilized to fit the relaxation modulus and the curves are plotted in Figure 16.

As shown in Figure 16, the relaxation modulus decreases versus time, in which the change rate of relaxation modulus is larger in the initial loading stage and the rate tends to be stable in the late loading stage. This observation is consistent with the stress relaxation characteristics of asphalt mortar. Besides, the relaxation modulus could be ranked from big to small as: F3 > F6 > F5 > F1 > F7 > F10 > F4 > F9 > F2 > F8, which is in good agreement with the stiffness modulus of the beam bending failure test. The higher the relaxation modulus, the larger the failure possibility, which suggests an adverse low-temperature cracking resistance of asphalt mortar prepared by manufactured sands. From the partial enlarged drawing during a period of 800 s to 1800 s, it can be seen that the relaxation modulus of asphalt mortars made by manufactured sands (i.e., F3, F5 and F6) are still in a clear declining stage, while the relaxation modulus of other groups with river sands trend to be stable.

(2) Influence of Fine Aggregate on Low-Temperature Viscoelastic Parameters

**Figure 17:**
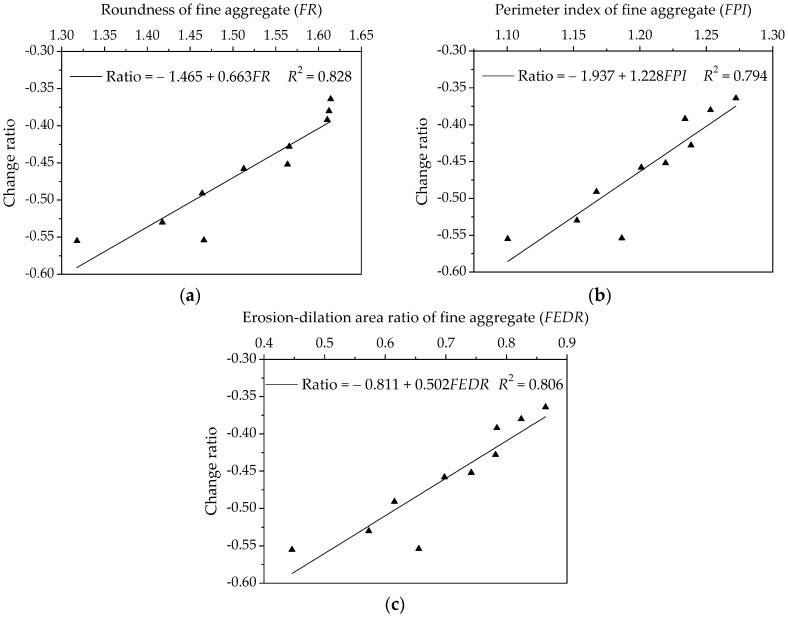
Influences of fine aggregate morphological characteristics of on change rate of relaxation modulus for asphalt mortars: (**a**) Shape (*FR*); (**b**) Angularity (*FPI*); and, (**c**) Texture (*FEDR*).

The change rate of relaxation modulus as an evaluating index is used to reflect the relaxation characteristics of asphalt mortar. A larger change rate of relaxation modulus is preferable, which means a shorter relaxation time and more rapid dissipation of stress. Observe that the relaxation modulus is nearly exponential decline, and then a linear fitting model could be obtained by using the logarithmic transformation. The slopes of linear fitting models were selected to characterize the change rate of relaxation modulus and regarded as the response variable depicted in Figure 17. The regression analysis of change rates versus morphological characteristics of fine aggregates is presented in Figure 17.

As shown in Figure 17, the correlation coefficient values *R*^2^ for three morphological characteristics are larger than 0.79, which demonstrates morphological characteristics have a certain correlation with change rate of relaxation modulus. There is a positive correlation among these parameters, that is, the change rate of relaxation modulus increases with morphological characteristics. It is clear that the more complex morphological characteristics the fine aggregate has, the smaller the change rate of relaxation modulus is. Thus, asphalt mortar prepared by manufactured sands has an adverse low-temperature cracking resistance.

### 4.3. Analysis for Coarse Aggregate Morphological Characteristics on Relaxation Properties of Asphalt Mixture

#### 4.3.1. Beam Bending Failure Test Results of Asphalt Mixture

**Figure 18:**
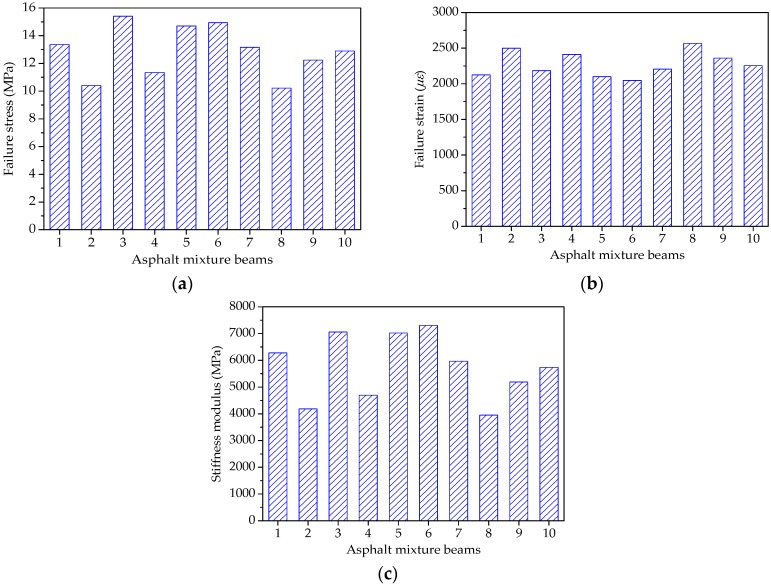
Failure results of asphalt mixture beams: (**a**) Failure stress; (**b**) Failure strain; and, (**c**) Stiffness modulus.

Figure 18 shows the bending failure stress, strain and stiffness modulus results of all the asphalt mixtures. The beam bending failure test of asphalt mixture was conducted using the same procedure and test condition with asphalt mortar. As shown in Figure 18a, the bending failure stress could be ranked from big to small, as follows: C3 > C6 > C5 > C1 > C7 > C10 > C9 > C4 > C2 > C8. The bending failure stress characterizes the ultimate load under the bending-tension state, and therefore manufactured stones can improve the bending failure strength of asphalt mixture. The bending failure strains for 10 groups of asphalt mixtures are ranked as C8 > C2 > C4 > C9 > C10 > C7 > C3 > C1 > C5 > C6, as shown in Figure 18b. Additionally, corresponding stiffness modulus are ranked as: C6 > C3 > C5 > C1 > C7 > C10 > C9 > C4 > C2 > C8. It is evident that asphalt mixtures with pebble have a larger bending failure strain and lower stiffness modulus when compared with asphalt mixture made by manufactured stones, which implies that pebble could improve the low-temperature ductility and cracking resistance of asphalt mixture. This is expected because pebble is more spherical with lower angularity values; the inlay effect in asphalt mixture becomes weaker while loaded, leading to smaller failure strength.

#### 4.3.2. Beam Bending Creep Test Results of Asphalt Mixture

(1) Strain Results of Low-Temperature Creep Test

**Figure 19:**
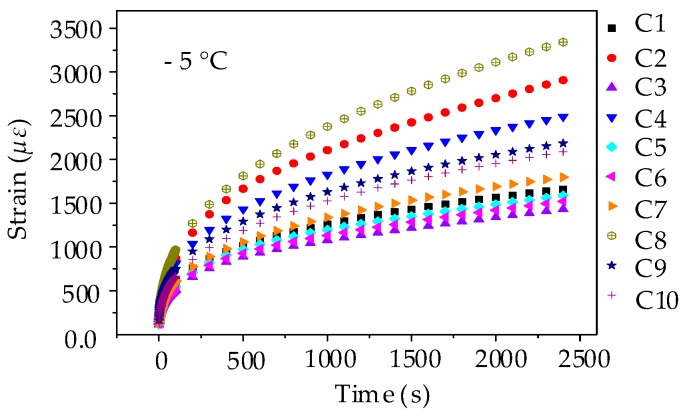
Creep strain-time curves of asphalt mixture beams.

The procedure of beam bending creep test for asphalt mixture was in accordance with asphalt mortar except for the loading time. The creep strains for 10 testing groups of asphalt mixtures are ranked as C8 > C2 > C4 > C9 > C10 > C7 > C1 > C5 > C6 > C3, as shown in Figure 19. This observation is mainly consistent with the creep results of asphalt mortar, in which asphalt mixture prepared by pebble (i.e., C8) has the largest strain, whereas asphalt mixture prepared by andesite stone (i.e., C3) has the smallest strain. Besides, the bending creep strain also shows a rising trend with pebble proportion.

**Figure 20:**
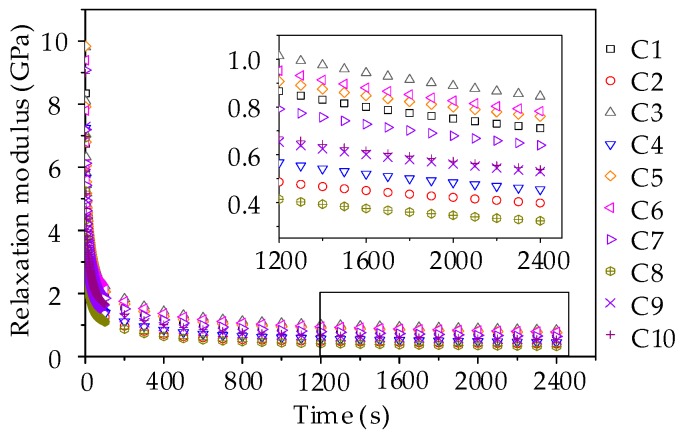
Relaxation modulus curve of asphalt mixtures.

Similarly, by fitting the creep strain results, the parameters of the Prony series model of relaxation modulus could be obtained and have the higher *R*^2^ values above 0.998, as listed in Table 9. The relaxation modulus curves were therefore constructed and plotted in Figure 20. The relaxation modulus could be ranked as: C3 > C6 > C5 > C1 > C7 > C10 > C9 > C4 > C2 > C8 and the relaxation modulus are still in a clear declining stage in the partial enlarged drawing. Regarding the comparison of relaxation modulus, it is worth noting that the relaxation modulus exhibits a declining trend with the content of pebble. Hence, it can be concluded that pebble could improve the low-temperature cracking resistance of asphalt mixture.

(2) Influence of Coarse Aggregate on Low-Temperature Viscoelastic Parameters

**Figure 21:**
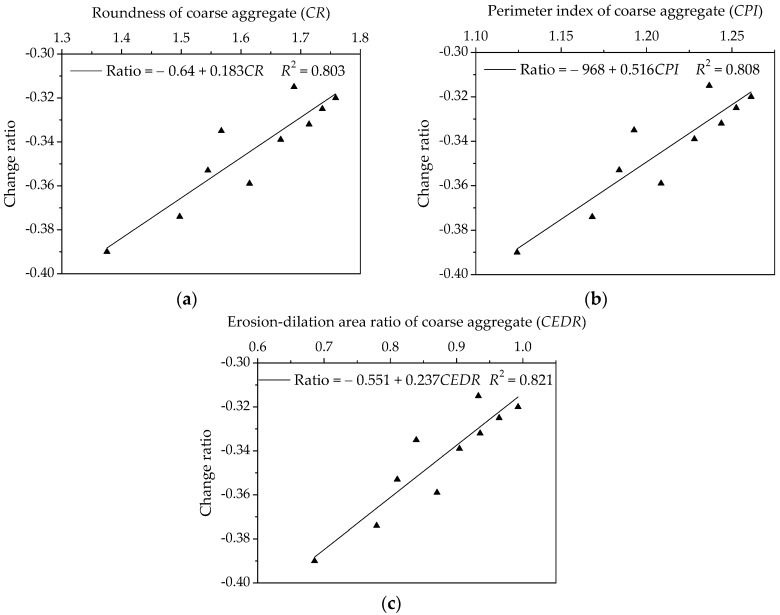
Influences of coarse aggregate morphological characteristics of on change rate of relaxation modulus for asphalt mixtures: (**a**) Shape (*CR*); (**b**) Angularity (*CPI*); and, (**c**) Texture (*CEDR*).

The influence of morphological characteristics of coarse aggregate on low-temperature relaxation characteristics are investigated by using linear regression, in which morphological characteristics (i.e., *CR*, *CPI* and *CEDR*) are regarded as independent variables and change rate of relaxation modulus through logarithmic transformation are response variables. The regression analysis results are presented in Figure 21. As shown in Figure 21, the correlation coefficient values *R*^2^ for three morphological characteristics are larger than 0.8, which demonstrates that aggregate morphological characteristics have a certain correlation with the change rate of relaxation modulus. There is a positive correlation among these parameters, thus, asphalt mixture prepared by manufactured stones has an adverse low-temperature cracking resistance.

In addition, it must be noted that the change rate range of relaxation modulus for asphalt mixture is lower when compared to asphalt mortar, in which the change rate range of relaxation modulus is 0.31 to 0.39 (in absolute value) for asphalt mixture, as shown in Figure 21, and the range for asphalt mortar is 0.36 to 0.56 (in absolute value), as shown in Figure 17. This is expected because the stress relaxation mainly relates to asphalt mortar rather than asphalt mixture. However, the proportions of aggregates in asphalt mixtures are basically the same except the coarse aggregates. Therefore, there is a relatively small difference for relaxation characteristics among asphalt mixtures, which also demonstrates a lower correlation with relaxation characteristics for coarse aggregates as compared to fine aggregates.

## 5. Optimization of Aggregate Morphological Characteristics for High- and Low-Temperature Viscoelasticity

The influences of morphological characteristics of fine and coarse aggregates on high and low temperature viscoelastic characteristics of asphalt mortar and mixture have been discussed above. It is evident that aggregates with complex morphological characteristics could improve the resistance to deformation at high temperature and have an adverse low-temperature cracking resistance. Consequently, it is necessary to optimize the aggregate morphological characteristics in asphalt mortar and mixture while considering high and low temperature viscoelastic characteristics.

### 5.1. Optimization for Morphological Characteristics of Fine Aggregate

In order to better optimize morphological characteristics of fine aggregate, the compression failure stress at 30 °C, retardation time and bending strain at −5 °C, and change rate of relaxation modulus are employed to characterize the high and low temperature viscoelastic characteristics, respectively. Based on the aggregate proportioning design by SLD method and corresponding test results, the regression analysis was conducted for the relationship between morphological characteristics and viscoelastic characteristics. The optimal values of single response for asphalt mortar were obtained firstly at different aggregate proportioning designs. Then, comprehensively considering high and low temperature viscoelastic characteristics, the aggregate proportioning design could be determined, i.e., basalt:andesite:river sand = 0:0.582:0.418, and the predictive values of multi-response as well as test results were also compared and listed in Table 10. As can be seen from the results, the relative errors are less than 4%, indicating a higher predictive accuracy. It could be concluded that asphalt mortar designed by the optimal aggregate proportion has the best high- and low-temperature viscoelastic characteristics, in which the morphological characteristics of fine aggregate are *FR* = 1.4909, *FPI* = 1.2005, and *FEDR* = 0.6896.

### 5.2. Optimization for Morphological Characteristics of Coarse Aggregate

As for the optimization of coarse aggregate, the compression failure stress at 50 °C, retardation time are selected as high-temperature viscoelastic indices and the bending strain at −5 °C, change rate of relaxation modulus are regarded as low-temperature viscoelastic indices. Based on the optimal values of single response, the aggregate proportion for asphalt mixture while considering high and low temperature viscoelastic characteristics was designed as: basalt:andesite:pebble = 0.508:0.050:0.442. Table 11 compares the predictive values of multi-response and test results for asphalt mixture with relative errors less than 6%. When the morphological characteristics of coarse aggregate are *CR* = 1.5666, *CPI* = 1.1918, *CEDR* = 0.828, asphalt mixture has the best high and low temperature viscoelastic characteristics.

## 6. Conclusions

This study evaluated the influences of aggregate morphological characteristics on the high- and low-temperature viscoelastic characteristics of asphalt mixtures. Based on SLD, three different types of aggregates were used to prepare asphalt mixtures and aggregates were characterized by three morphological indices. Accordingly, the viscoelastic characteristics of asphalt mortar and mixture were evaluated based on a uniaxial compression static creep test and beam bending creep test. Based on the analysis results, the following conclusions could be drawn:

(1) Aggregate morphological characteristics were proved to strongly correlate with the viscoelastic properties of asphalt mortar and mixture. Asphalt mortar consisted of fine aggregate demonstrated a stronger correlation with the creep characteristics of the mix.

(2) Results showed that the resistance to deformation of asphalt mortar and mixture increases with increasing aggregate morphological characteristics, especially coarse aggregates, could effectively enhance the resistance to deformation of asphalt mixture due to the skeleton effect.

(3) Test analysis results indicated that aggregate morphological characteristics presented high correlations with the high-temperature creep and low-temperature relaxation characteristics. The observations also showed that with morphological characteristics increasing, the capacity of deformation recovery and anti-deformation could be improved, whereas the low-temperature cracking resistance would have an adverse influence.

(4) Based on the comprehensive consideration of high- and low-temperature viscoelastic properties, the aggregate proportion was optimized to obtain the more appropriate aggregate morphological characteristics for asphalt mixtures and the results also showed good agreement between predictive and experimental values. The optimization for aggregate morphological characteristics should be considered in asphalt mixture design in order to improve the performances of asphalt pavement.

## Figures and Tables

**Table 1:** Physical properties of base asphalt AH-90.

Property	Unit	Measurement	Technical Criterion
Penetration @ 25 °C, 100 g, 5 s	0.1 mm	90	80~100
Softening point	°C	42.6	≥42
Ductility @ 15 °C, 5 cm/min	cm	195.2	≥100
Density @ 15 °C	g/cm^3^	1.014	-
*After TFOT*			
Mass loss	%	0.37	±0.8
Penetration ratio @ 25 °C	%	59	≥54

**Table 2:** Properties of limestone powder.

Property	Specific Gravity	Specific Surface Area	Hydrophilic Coefficient	Main Composition
Value	2.652	0.886	0.80	CaCO_3_
Unit	g/cm^3^	m^2^/g	-	-

**Table 3:** Apparent densities of coarse and fine aggregates (Unit: g/cm^3^).

Size (mm)	13.2	9.5	4.75	2.36	1.18	0.6	0.3	0.15	0.075
Basalt	2.782	2.774	2.770	2.758	2.713	2.720	2.699	2.647	2.700
Andesite	2.785	2.853	2.729	2.717	2.658	2.701	2.639	2.645	2.648
Pebble/River Sand	2.656	2.644	2.645	2.636	2.624	2.606	2.635	2.698	2.597

**Table 4:** Properties of coarse aggregate.

Property	Natural Bulk Density (%)	Tapped Bulk Density (%)	Wearing Value (%)
Basalt Stone	1.511	1.636	10.18
Andesite Stone	1.502	1.635	11.26
Pebble	1.536	1.640	23.64

**Table 5:** Experimental proportion design based on simplex lattice design (SLD).

Mix Component	Sample Group									
	1	2	3	4	5	6	7	8	9	10
**Basalt**	0.167	0.167	0.000	0.500	1.000	0.500	0.667	0.000	0.000	0.333
**Andesite**	0.667	0.167	1.000	0.000	0.000	0.500	0.167	0.000	0.500	0.333
**Pebble/River Sand**	0.167	0.667	0.000	0.500	0.000	0.000	0.167	1.000	0.500	0.333

**Table 6:** Each component content of asphalt mixes.

Component	Proportion	Aggregate	Filler	Asphalt	Air Void
Asphalt Mixture	Mass (%)	89.5	5.7	4.8	-
	Volume (%)	79.3	5.2	11.5	4
Asphalt Mortar	Mass (%)	74.1	14.3	11.6	-
	Volume (%)	59.8	11.6	24.6	4

**Table 7:** Composite morphological index of fine and coarse aggregates.

Group	1	2	3	4	5	6	7	8	9	10
**Asphalt Mortar**	**F1**	**F2**	**F3**	**F4**	**F5**	**F6**	**F7**	**F8**	**F9**	**F10**
***FR***	1.5658	1.4175	1.6143	1.4641	1.6103	1.6123	1.5638	1.3178	1.4661	1.5127
***FPI***	1.2385	1.1526	1.2723	1.1673	1.2340	1.2532	1.2194	1.1005	1.1864	1.2011
***FEDR***	0.7821	0.5729	0.8645	0.6151	0.7842	0.8244	0.7419	0.4461	0.6553	0.6976
**Asphalt Mixture**	**C1**	**C2**	**C3**	**C4**	**C5**	**C6**	**C7**	**C8**	**C9**	**C10**
***CR***	1.6889	1.4974	1.7586	1.5447	1.7140	1.7363	1.6666	1.3755	1.5671	1.6144
***CPI***	1.2367	1.1682	1.2612	1.1840	1.2438	1.2525	1.2280	1.1242	1.1927	1.2085
***CEDR***	0.9329	0.7794	0.9928	0.8106	0.9355	0.9642	0.9043	0.6857	0.8392	0.8705

**Table 8:** Fitting results of Prony series for asphalt mortars.

Group	F1	F2	F3	F4	F5	F6	F7	F8	F9	F10
*E*_∞_ (GPa)	7.67	10.79	8.74	7.61	8.62	8.61	7.45	9.58	6.50	9.25
*E*_1_ (GPa)	4.28	2.72	5.72	3.79	5.00	5.57	4.24	2.84	4.03	3.47
*E*_2_ (GPa)	19.84	24.74	24.00	18.32	24.05	23.99	19.43	23.47	19.15	22.71
*E*_3_ (GPa)	1.77	1.10	2.51	1.46	2.11	2.38	1.70	1.10	1.44	1.45
*E*_4_ (GPa)	0.52	0.25	0.90	0.32	0.86	0.89	0.45	0.23	0.29	0.42
*E*_5_ (GPa)	0.17	0.06	0.37	0.10	0.23	0.29	0.14	0.05	0.06	0.12
*R* ^2^	0.9991	0.9985	0.9989	0.9994	0.9995	0.9987	0.9992	0.9996	0.9998	0.9997

**Table 9:** Fitting results of Prony series for asphalt mixtures.

Group	C1	C2	C3	C4	C5	C6	C7	C8	C9	C10
*E*_∞_ (GPa)	19.66	11.31	30.16	18.80	27.41	32.27	22.70	20.15	18.09	23.68
*E*_1_ (GPa)	28.14	8.52	26.22	25.60	30.06	23.55	31.49	25.85	25.21	32.00
*E*_2_ (GPa)	10.06	34.84	12.52	7.96	11.62	12.21	10.31	7.15	8.36	9.76
*E*_3_ (GPa)	5.17	4.05	6.14	3.73	5.80	5.74	5.00	3.05	4.09	4.51
*E*_4_ (GPa)	2.67	1.31	2.87	1.79	2.43	2.84	2.47	1.37	2.03	2.14
*E*_5_ (GPa)	0.85	0.46	1.05	0.50	0.96	0.93	0.73	0.34	0.60	0.59
*R* ^2^	0.9999	0.9996	0.9985	0.9994	0.9996	0.9989	0.9992	0.9994	0.9999	0.9993

**Table 10:** Optimal value of multi-response for asphalt mortar.

Property	High-Temperature Property		Low-Temperature Property	
	Failure Stress (MPa)	Retardation Time (s)	Bending Strain	Change Rate
Prediction	2.98	150	5393	−0.523
Experiment	3.06	145	5587	−0.531
Relative Error (%)	2.7	−3.3	3.6	1.5

**Table 11:** Optimal value of multi-response for asphalt mixture.

Property	High-Temperature Property		Low-Temperature Property	
	Failure Stress (MPa)	Retardation Time (s)	Bending Strain	Change Rate
Prediction	3.15	81.6	2330	−0.356
Experiment	3.21	86.5	2438	−0.349
Relative Error (%)	1.9	6	4.6	−2
